# Construction and Analysis of circRNA-miRNA-mRNA Molecular Regulatory Networks During Herba *Gelsemium elegans* Intoxication

**DOI:** 10.3389/fphar.2019.01217

**Published:** 2019-10-17

**Authors:** Yinghao Wang, Shuisheng Wu, Ce Yang, Hanyun Gao, Hongmin Yu, Xuehua Lu, Shugang Yuan

**Affiliations:** ^1^College of Pharmacy, Fujian University of Traditional Chinese Medicine, Fuzhou, China; ^2^Institute of Materia Medica, Fujian Medical Science Research Institute, Fuzhou, China

**Keywords:** circRNA-miRNA-mRNA, microarray, molecular regulatory networks, intoxication, *Gelsemium elegans* (Gardner & Champ.) Benth

## Abstract

*Gelsemium elegans* (Gardner & Champ.) Benth. (GE) has therapeutic effects for pain and malignant tumors but also has high toxicity. Its mechanism of toxicity has not yet been fully clarified, thus limiting its application. Meanwhile, evidence has shown that circRNAs are closely related to the progression of disease. However, very little is known about their expression profiles during intoxication. In this paper, circRNA/mRNA microarrays were respectively performed to detect their expression profiles in mice with acute GE intoxication versus normal controls. CircRNAs were verified by qRT-PCR in subsequent experiments. A regulation pattern of circRNA→miRNA→mRNA was deduced based on intersection analysis of circRNA/mRNA microarrays. The results revealed circRNAs (143) and mRNAs (1,921) were significantly expressed during intoxication. Most of the circRNAs were exonic, and most distributions in chromosomes were transcribed from chr1, chr2, chr7, and chr11. Furthermore, dysregulated expression of mmu-circRNA-013703 and mmu-circRNA-010022 was verified. Then a circRNA-targeted miRNA-mRNA co-expression network was constructed. The network map contained 2 circRNAs, 52 miRNAs, and 752 mRNAs. GO & KEGG analysis further predicted that mmu-circRNA-013703 and mmu-circRNA-010022 may participate in cellular survival/demise-related, neuron/synapse-related, and channel-related pathways. Based on functional modules analysis, a new network was formed, in which mmu-circRNA-013703 VS mmu-miR-361-3p linked to most mRNAs. Most of these mRNAs were known to be involved in the aforementioned functional module. This indicated that mmu-circRNA-013703 functioned as a sponge of miRNAs to regulate the more comprehensive circRNA-miRNA-mRNA co-expression network. Our approach revealed a landscape of dysregulated circRNA-miRNA-mRNA and may be valuable for the identification of new biomarkers during intoxication.

## Introduction

*Gelsemium elegans* (Gardner & Champ.) Benth. (GE), belonging to the Loganiaceae family of plants, is commonly known as a deadly poison. However, an increasing number of studies have shown that it has therapeutic effects for pain, malignant tumors, psoriasis, rheumatic arthritis, and immune function (Rujjanawate et al., 2003; Xu et al., 2012a; Xu et al., 2012b). Despite the benefits of GE preparations to human health, they are not used extensively due to their high toxicity. Although GE toxicity has been evaluated in animal models (Rujjanawate et al., 2003), no scientific investigations have been reported, to the best of our knowledge, with regard to the mechanism of toxicity. Thus, the application of GE has been limited to a great extent. In our prior research, we found that GE toxicity is associated with hepatic microsomal enzymes (Wang et al., 2018). However, recent progress in molecular biology has given us the opportunity for further study.

In recent years, accumulating evidence has shown that non-coding RNAs (ncRNAs) are involved in various diseases (Guttman and Rinn, 2012; Castel and Martienssen, 2013; Meng et al., 2017). Circular RNAs (circRNAs), novel members of the ncRNA family that are widespread and substantial within transcriptomes (Memczak et al., 2013), have drawn the attention of many researchers (Hansen et al., 2013; Jeck and Sharpless, 2014; Gao et al., 2015). CircRNAs have been confirmed to be highly stable due to their covalently closed loop structures without 5′ end caps or 3′ poly (A) tails (Liang and Wilusz, 2014). As a result of their substantial stability and specificity within certain tissues, circRNAs could be perfect molecular biomarkers for many diseases (Rybak-Wolf et al., 2015; Chen et al., 2017b). Recent, developments in microarray techniques and high-throughput sequencing technologies have been made, enabling investigators to conduct comprehensive analyses on the functions and profiles of circRNAs (Ashwal-Fluss et al., 2014; Conn et al., 2015). CircRNAs may function as microRNAs (miRNAs) sponges, form RNA-protein complexes and regulate targeted gene splicing and transcription (Memczak et al., 2013). Accordingly, circRNAs have been hypothesized to regulate occurrence and development of disease by sequestering disease-specific miRNA (Chen et al., 2016). To date, numerous circRNAs have been successfully identified in various organisms and some have been found to be association with the progression of human diseases (Wang et al., 2016a; Zheng et al., 2016).

However, very little is yet known about the role of circRNAs in drug intoxication. There have been no reports on the expression profiles of circRNAs during intoxication. Therefore, we focused on the intoxication-associated mechanism partly because of its importance in clinical safety. We hoped that changes in circRNAs during intoxication would give our data wider relevance. For this purpose, circRNAs/mRNAs microarrays were performed to examine their expression profiles under intoxication and normal conditions. Furthermore, GO and KEGG pathways were analyzed and a co-expression network of latent targeted relationships was structured according to the mRNAs with altered expression levels under abnormal conditions in negative or positive correlation with changing circRNAs.

## Materials and Methods

### Chemicals and Reagents

Rnase R and RNasin Inhibitor were supplied by Epicenter (Madison, WI). SuperScript™ III Reverse Transcriptase, TRIzol, 5×RT Buffer solution, RNase-Free water, and RNase-Free glycogen were obtained from Invitrogen Life Technologies (Carlsbad, CA). dNTP Mix was supplied by HyTest Ltd. (Turku, Finland). Ultrapure water was supplied by a Milli-Q water purification system (Bedford, MA). All other chemicals and solvents used were of at least analytical grade.

### Chemical Profiles of Extract

**Figure 1:**
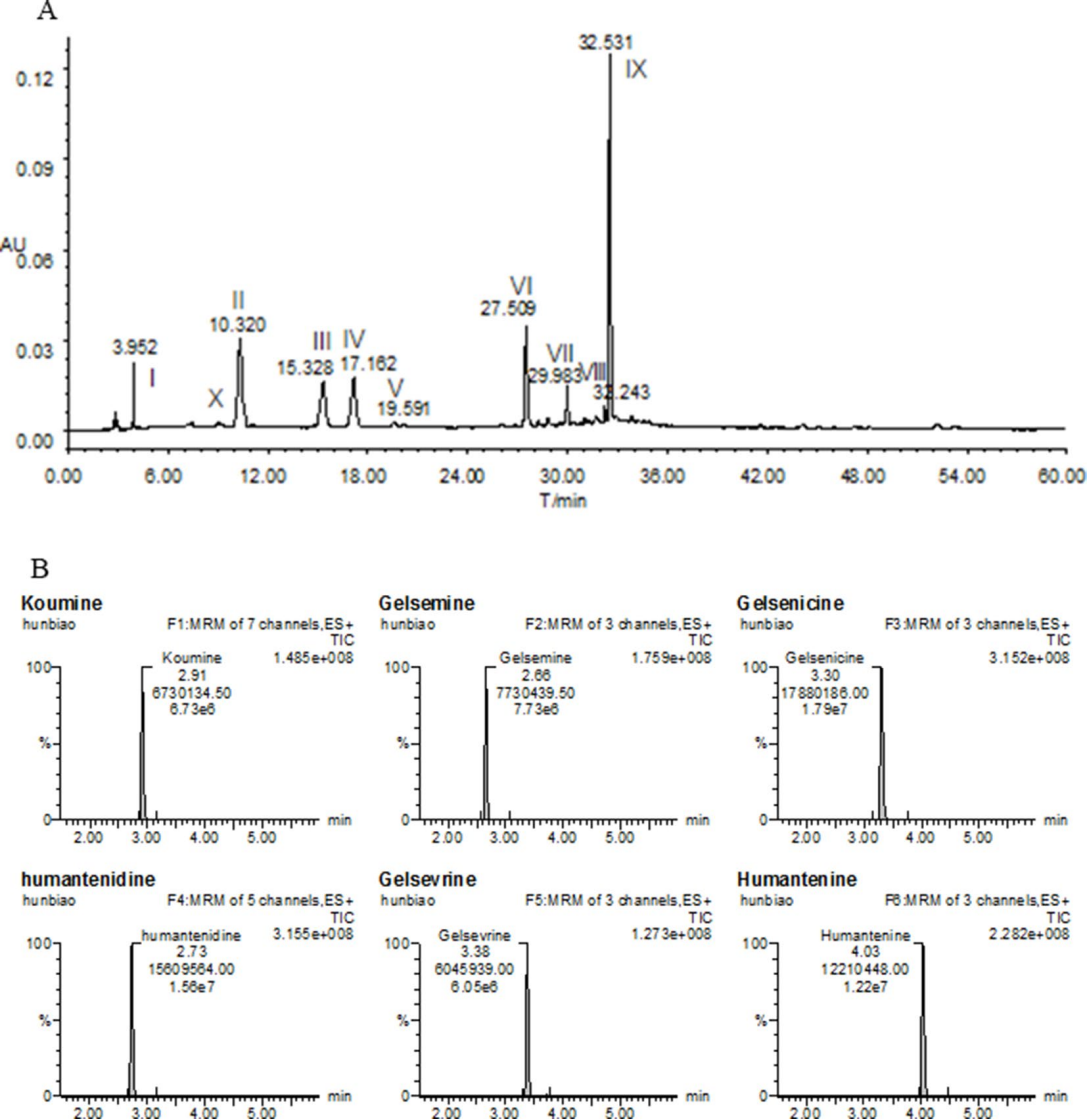
Chemical profiles of **(A)** HPLC and **(B)** LC-MS/MS of most of the ingredients in GE extract. I, Humantenidine; II, Koumine; III, Gelsenicine; IV, Gelsevirine; VI, Humantenine; X, Gelsemine; V, VII, VIII and IX, unknown compounds.

Fresh roots of GE were obtained from cultivable sources in the Fujian province of China (H: 518m, LA: 25’’ 47’ 0528N, LO: 118’’ 40’ 4066E). It was authenticated by Prof. Shuisheng Wu from Fujian University of Traditional Chinese Medicine (FJTCM). The roots were sliced, dried, and ground. The extraction process of the dried powder was identical to that detailed earlier (Wang et al., 2017a; Wang et al., 2018). Concentration was 0.05 g/ml and filtered and evaporated under reduced pressure to obtain GE dichloromethane extract with typical yields of 0.67 ± 0.071%. The high-performance liquid chromatography profiles of the extract was performed as previously described (Wang et al., 2015; Wang et al., 2017a). Chemical composition identification of the extract was achieved by LC-ESI-MS/MS method as previously described (Wang et al., 2015; Wang et al., 2017a). The chemical profiles were shown in **Figure 1A**. The major chemical constituents were indole alkaloids, including koumine, humantenmine, gelsemine, gelsenicine, humantenine, and gelsevirine (see **Figure 1B**).

### Animals, Treatments, and Tissue Preparation

All experimental procedures complied with international ethical guidelines and the Guide for the Care and Use of Laboratory Animals (National Institutes of Health). The animal protocol was approved by the Ethics Committee of Fujian University of Traditional Chinese Medicine [FJTCM (2018) Ethics Review No. 018]. A total of 12 healthy ICR male mice (18–22 g) were provided by Shanghai SLAC Laboratory Animal Co., Ltd. (Shanghai, China). The mice were randomly divided into control and GE administration groups. They were housed in controlled environments at a constant temperature (25 ± 1°C) under a 12:12 h light-dark cycle and allowed free access to feedstuff and distilled water. After acclimatizing for one week, the administration group was intragastrically treated with GE at a dose (0.40 g/kg) previously considered toxic and routinely used (Wang et al., 2016b). The control group was given the same volume of normal saline. After 2 h of observation, the mice were sacrificed and their livers were immediately excised, washed with pre-cooled normal saline, frozen in liquid nitrogen, and then stored at −80°C until further experiments.

### Total RNA Extraction and Quality Control

Total RNA isolation was performed by TRIzol reagent according to the manufacturer’s instructions. The concentration of extracted RNAs was determined using a NanoDrop 2000C spectrophotometer (Thermo Fisher Scientific, Waltham, MA), and their integrities were tested by electrophoresis on a denaturing agarose gel. The extracted RNAs were stored at −80°C for further analyses.

### CircRNA and mRNA Microarray Detection

Three extracted RNAs from each group were digested with Rnase R to remove linear RNAs and enrich the circRNAs. Thereafter, another extracted RNAs and the three circRNAs were amplified and transcribed into fluorescent cRNA respectively. The labeled mRNAs and circRNAs were separately hybridized onto the Whole Mouse Genome Oligo Microarray (4×44K, Agilent Technologies, Santa Clara, CA) and the Arraystar Mouse circRNA Array V2 (8×15K, Arraystar Inc., Rockville, MD). The whole mouse genome oligo/circRNA array was one color. The specific model was microarray v2. The GEO database links used are as follows: https://www.ncbi.nlm.nih.gov/geo/query/acc.cgi?acc=GPL21826. After washing the slides, the microarrays were scanned using the Agilent Scanner G2505C (Agilent Technologies).

### Microarray Data Collection and Visualization Analysis

The acquired microarray images were analyzed by Agilent Feature Extraction software (version 11.0.1.1) to extract the raw data. Low-intensity filtering was carried out in the subsequent quantile normalization of the raw data by R software package (version 3.1.2). A Box-plot was used to visualize the distribution of the normalized intensities from all datasets. After quantile normalization, circRNAs in at least 3 out of 9 samples and mRNAs in at least 6 out of 12 samples tagged “P” or “M” (“all targets value”) were chosen for extensive data analysis. P values <0.05 and fold changes greater than 1.5 between the two groups was used to identify differential expression of mRNAs/circRNAs. A Volcano Plot was adopted to visualize the differentially expressed mRNAs/circRNAs between the two groups. Hierarchical clustering was further used to display the distinguishable expression profiles of mRNAs/circRNAs. Finally, the types of circRNAs and their distributions among chromosomes were analyzed.

### Validation of Candidate circRNAs

Because circRNAs, whose targeted miRNAs were predicted in microarray analysis, and the fold change and p-value of expression were confirmed to be involved in the worsening of intoxication in previous findings, 5 circRNAs (mmu-circRNA-013703, mmu-circRNA-008436, mmu-circRNA-010022, mmu-circRNA-23433, and mmu-circRNA-40996) were chosen for validation purposes. Total RNA extraction and quality control was the same as the above-mentioned. Subsequently, reverse transcriptase reactions were conducted in a 20-μl reaction volume, including 3.0 µg of total RNA, 0.5 µg Random (N9), 1.6 µl dNTPs Mix (2.5mM), 13.5 µl RNase-Free water, 4 µl 5× First-Strand Buffer, 1 µl 0.1M DTT, 0.5 µl RNase Inhibitor and 1 µl SuperScript III RT. Each cDNA of 2.0 µl was amplified *via* PCR with specific primers. The specific primers were as follows: F 5′-CGCCAAAGAACAGAGATACCAG-3′ and R 5′-TACCGTTTATTTCACCATAGCCT-3′ for mmu-circRNA-013703; F 5′-ACACGGAGATCCCAGAAGGAA-3′ and R 5′-CGCAGTCACGTCTTCAGCAGT-3′ for mmu-circRNA-008436; F 5′-CTGAGTACCCCTCTGTCTACGCA-3′ and R 5′-GTAGACAATGGTGTACGGCGG-3′ for mmu-circRNA-010022; F 5′-GTAGACCGTTTAGCTCGAAAGCC-3′ and R 5′-TCATGACGACGACATCAGCTGT-3′ for mmu-circRNA-23433; F 5′-AACTTTACAGGCTGTTAGTCCCC-3′ and R 5′-AAATACCCTCCTCCTTCTCATCG-3′ for mmu-circRNA-40996; and F 5′-CACTGAGCAAGAGAGGCCCTAT-3′ and R 5′-GCAGCGAACTTTATTGATGGTATT-3′ for GAPDH (M). The PCR conditions were initiated at 95°C for 10 min, followed by 95°C (10 s) and 60°C (60 s) for a total of 40 cycles. The relative expression levels of circRNAs were calculated using the 2^-ΔΔCt^ method. All samples were analyzed by statistical methods before and after contrast.

### Construction of circRNA-miRNA-mRNA Co-Expression Network

To find potential circRNA-targeted miRNA interactions, the targeted miRNAs were predicted utilizing Arraystar’s miRNA target prediction software which is based on TargetScan (http://www.targetscan.org) (Enright et al., 2003) and miRanda (http://www.miranda.org/) (Pasquinelli, 2012). To further focus on the targeted profile of miRNAs, the top 5 putative targeted miRNAs were identified for each circRNA based on miRNA binding site and AU site. The putative targeted genes of these miRNAs were identified using Targetscan. The putative targeted genes and the differentially expressed genes obtained by microarray analysis were contrasted and analyzed by Venny (https://bioinfogp.cnb.csic.es/tools/venny/). Accordingly, a regulation pattern of circRNA→miRNA→mRNA was deduced based on the aforementioned intersection analysis. The differentially expressed circRNAs, miRNAs, and mRNAs were analyzed *via* Pearson’s correlation coefficient. The test calculates the P-value by using the following formula (Li et al., 2014). Strong correlation with P < 0.05 was a criterion for using the RNA for network construction. Cytoscape (www.cytoscape.org) was applied to construct the potential map of the circRNA-miRNA-mRNA co-expression network.

$$P=\sum_{i=c}^{\min(K,n)}\frac{\left(\begin{array}{c} K\\ i \end{array}\right)\left(\frac{N-K}{n-i}\right)}{\left(\begin{array}{c} N\\ n \end{array}\right)}$$

where N is the total number of miRNAs used to predict targets; K is the number of miRNAs that interact with the chosen gene of interest; n is the number of miRNAs that interact with the candidate ceRNA of the chosen gene.

### GO & KEGG Pathway Analysis and Functional Module Construction

The functions of the circRNAs were predicted by annotation of GO function and the KEGG pathway of targeted genes in the circRNA-miRNA-mRNA association. GO analysis (http://www.geneontology.org) was applied to reveal the roles of the targeted genes in terms of molecular functions (MF), cellular components (CC), and biological processes (BP). KEGG pathway analysis was used to annotate the functions and interactions of the targeted genes *via* the Database for Annotation, Visualization, and Integrated Discovery (https://david.ncifcrf.gov/home.jsp). P-values were adopted to test the reliability of the results. Lower p-values indicated a higher significance GO Term/Pathway (P-value cut-off 0.05). Enrichment bubble diagrams were then created to explore the GO Term/Pathway for significant differences in the co-expression of genes. Finally, the functional roles of the significantly different co-expressed genes were cataloged to draw a new functional module network.

## Results

### Validation of the Extracted RNA Quality and the Primers Target Specificity

The O.D. A260/A280 ratio was between 1.8 and 2.0, and the O.D. A260/A230 ratio was over 1.8. The 18S and 28S bands were intense and sharp, and the 5S band was present. The melt curve plots were all unimodal, indicating that the primers were target specificity (**Supplementary Figure 2**).

### Differential circRNA and mRNA Expression Profiling During GE Poisoning

**Figure 2:**
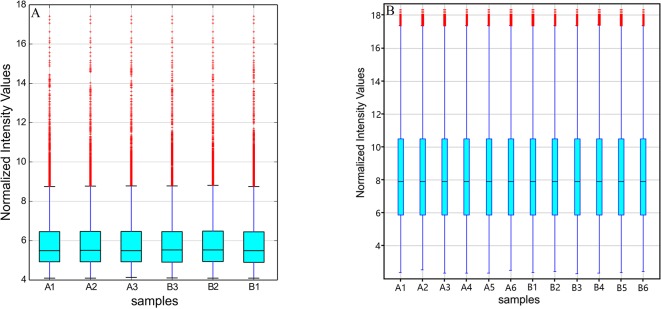
Box plots of **(A)** circRNAs and **(B)** mRNAs. Box plots compared the distributions of expression values for the tested samples after normalization (A1-6 for control and B1-6 for GE tissues).

The original expression values of circRNAs/mRNAs microarray data were standardized between the GE and control groups. Box plots were adopted to rapidly visualize the intensity distributions from all datasets after normalization (**Figure 2**). The results showed that the intensity distribution of log2 ratios was similar among the tested samples.

After the analysis of standardized microarray data, 143 circRNAs and 1,921 mRNAs were found to be significantly expressed during GE poisoning (P < 0.05). Among them, 22 circRNAs and 1,105 mRNAs were significantly upregulated. Moreover, 121 circRNAs and 816 mRNAs were significantly downregulated. It suggested that circRNA might play an important role in acute GE poisoning. According to related reports of intoxication and the results of P-value & fold change, the six representative circRNAs (3 up and 3 down) of significant difference are shown in **Table 1**. The rest are shown in **Supplementary Table 1**.

**Table 1:** Three significantly up/downregulated circRNAs.

circRNA	Regulation	*P*-value	Fold change	circRNA_type	Chromosome	Best_transcript	Gene Symbol
mmu_circRNA_39097	up	0.00042	1.53	intronic	chr5	ENSMUST00000112695	Zfp644
mmu_circRNA_40996	up	0.0069	1.77	exonic	chr6	NM_144920	Plekha5
mmu_circRNA_008286	up	0.0033	2.72	exonic	chr13	NM_028012	Xrcc4
mmu_circRNA_008614	down	0.017	3.43	exonic	chr9	ENSMUST00000183955	Mlip
mmu_circRNA_008436	down	0.0094	1.99	exonic	chr11	NM_001038609	Mapt
mmu_circRNA_010022	down	0.037	1.73	sense overlapping	chr19	NM_013541	Gstp1

**Figure 3:**
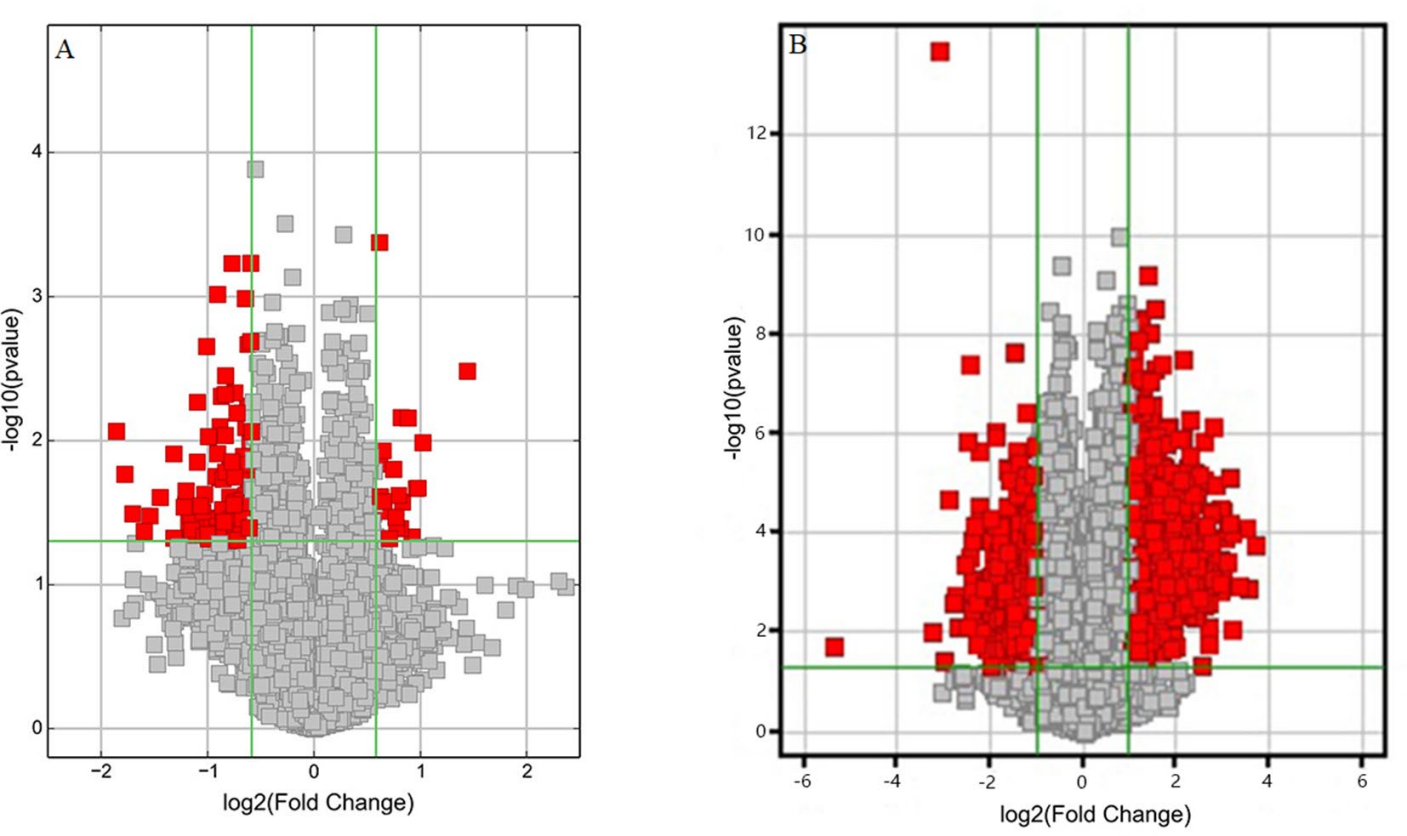
Volcano plots of differentially expressed **(A)** circRNAs and **(B)** mRNAs. Red points represent the differentially expressed circRNAs/mRNAs with statistical significance (Fold change > 1.5 and P < 0.05). Log2 (Fold change) < 0, downregulated transcripts; log2 (Fold change) > 0, upregulated transcripts.

Volcano plots were drawn to visualize the standardized expression of circRNAs and mRNAs between the GE and control groups. The red points in **Figure 3** represent differentially expressed circRNAs and mRNAs with statistical significance (P < 0.05). Upregulated and downregulated RNAs could be distinguished intuitively. The differential expression levels of circRNAs and mRNAs were the focus of subsequent research.

**Figure 4:**
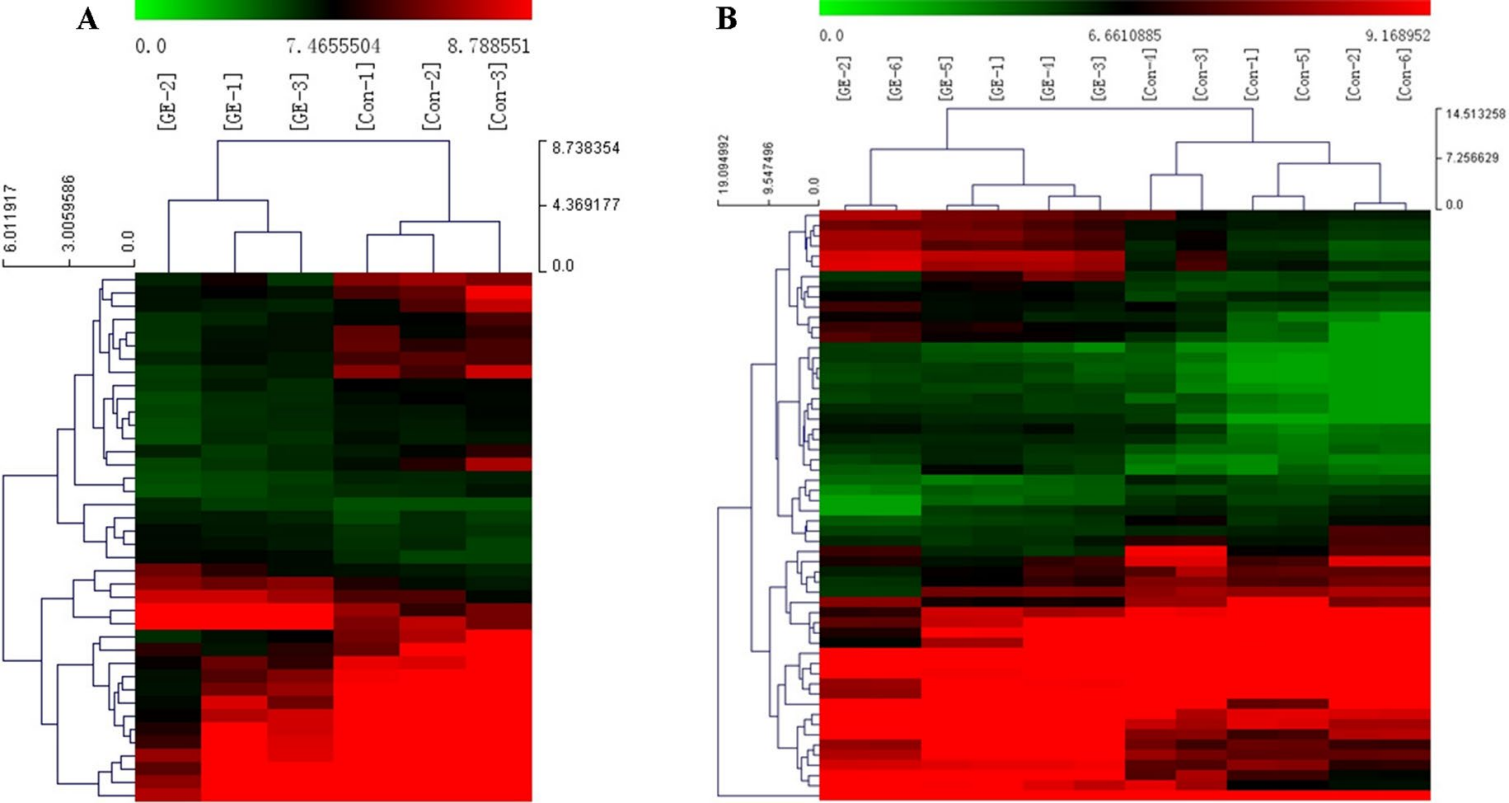
Cluster analysis of differentially expressed **(A)** circRNAs and **(B)** mRNAs. Red represents high expression levels; green represents low expression levels; each row represents a circRNA/RNA; each column represents the expression profile of a tissue sample.

A cluster analysis based on hierarchical clustering was analyzed and generated for the differentially expressed circRNAs and the differentially expressed mRNAs. Distinguishable circRNAs/mRNAs expression profiles from the samples are shown in **Figure 4**. The results suggested that circRNAs/mRNAs had differential expression patterns in the GE group in contrast with the control group.

### Types of circRNAs and Their Distribution on Chromosomes

**Figure 5:**
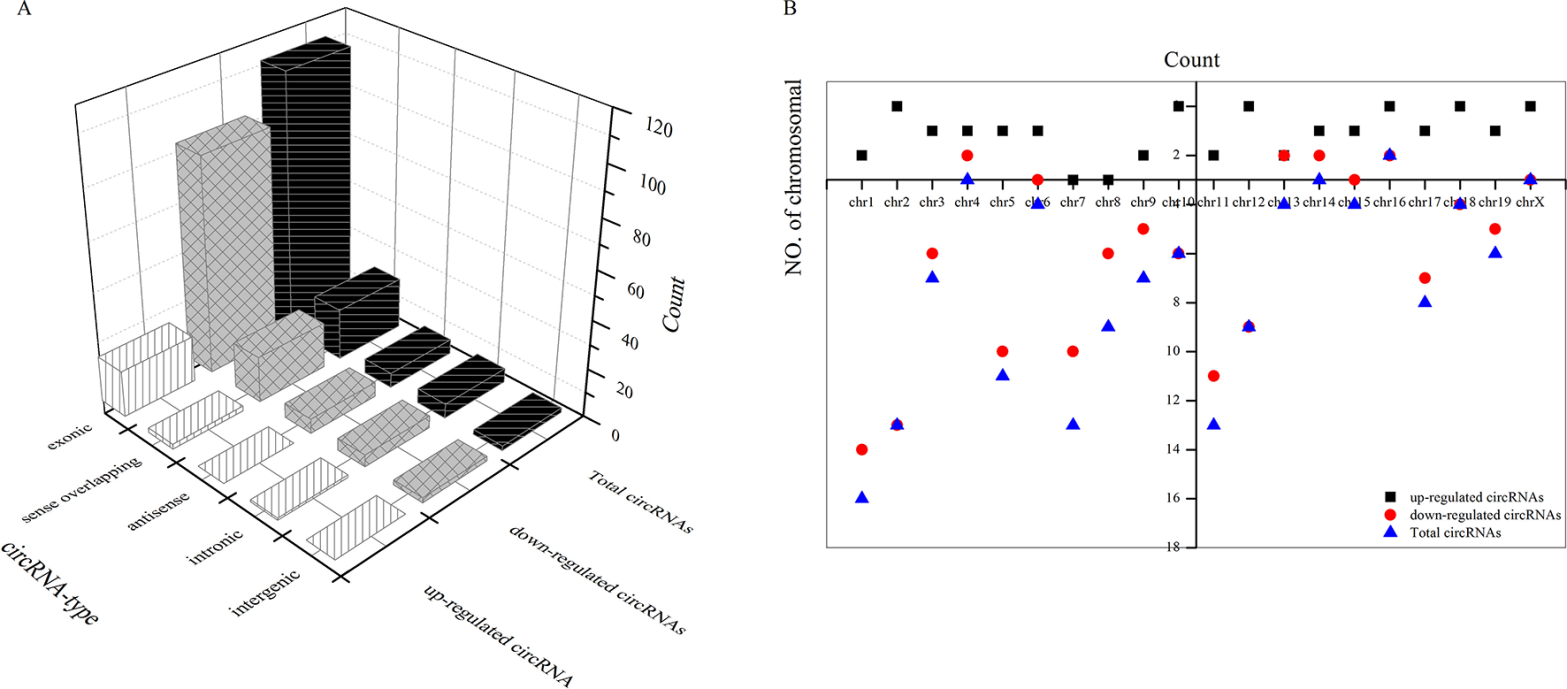
Types of differential circRNAs and their distribution on chromosomes. **(A)** types of differential circRNAs, **(B)** circRNAs distribution on chromosomes.

As shown in **Figure 5A**, most differentially downregulated circRNA were exonic, followed by sense overlapping. The results of upregulated circRNA were similar to those of downregulated circRNA. Moreover, the distributions of the circRNAs among chromosomes indicated that many circRNAs were transcribed from chr1, chr2, chr5, chr7, and chr11 but rarely from chr4, chr14, chr16, and chrX (**Figure 5B**).

### Regulation of circRNA-miRNA

**Figure 6:**
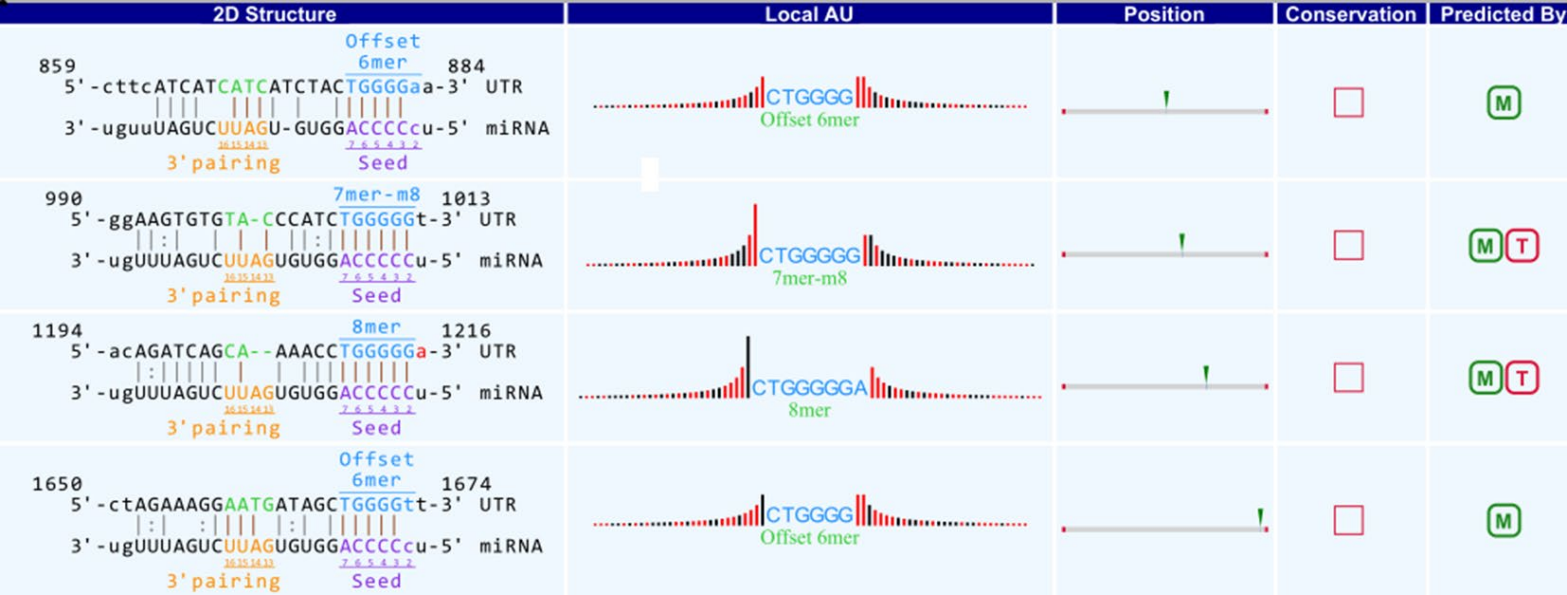
Binding site of mmu_circRNA_013703 and mmu-miR-361-3p. The upper part is the sequence at the junction of circRNA and the lower part is the sequence of miRNA.

A bioinformatics prediction was performed for the targeted miRNAs of differentially expressed circRNAs. The top 5 miRNAs with the best matching values were selected for each circRNA. A total of 532 miRNAs were bound to the 143 circRNAs with different expression levels. The targeted miRNAs of 5 representative circRNAs are shown in **Table 2**. The binding site, prediction criterion and AU site of circRNA association with targeted miRNA are shown in **Figure 6**, and **Supplementary Figure 1** and **4**, taking as an example mmu-circRNA- 013703 VS mmu-miR-361-3p. The results indicated that some miRNAs were bound by one circRNA, and other miRNAs were bound by two or more circRNAs. This implied that different circRNAs were connected *via* miRNAs.

**Table 2:** CircRNAs and their targeted miRNAs.

CircRNAs	miRNAs				
	MRE1	MRE2	MRE3	MRE4	MRE5
mmu_circRNA_008614	mmu-miR-5110	mmu-miR-504-3p	mmu-miR-185-5p	mmu-miR-7012-5p	mmu-miR-3154
mmu_circRNA_008436	mmu-miR-1934-5p	mmu-miR-504-5p	mmu-miR-6996-3p	mmu-miR-5626-3p	mmu-miR-28b
mmu_circRNA_010022	mmu-miR-326-5p	mmu-miR-328-5p	mmu-miR-6971-5p	mmu-miR-3078-3p	mmu-miR-7023-5p
mmu_circRNA_013703	mmu-miR-361-3p	mmu-miR-329-5p	mmu-miR-7065-3p	mmu-miR-1224-5p	mmu-miR-6989-3p
mmu_circRNA_23433	mmu-miR-7242-5p	mmu-miR-3099-5p	mmu-miR-467f	mmu-miR-3057-3p	mmu-miR-6393

### Validation of Candidate circRNAs

**Figure 7:**
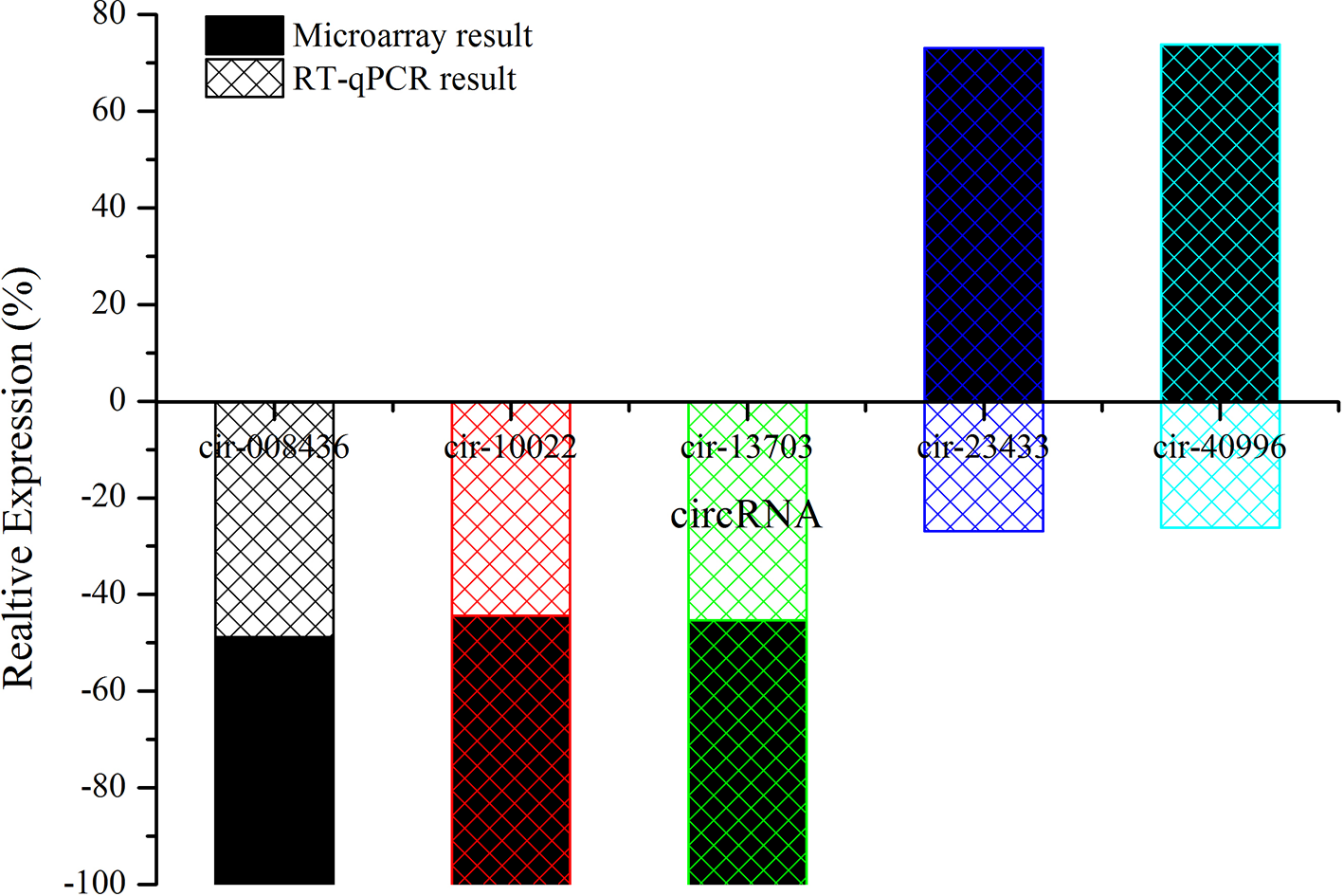
The contrast between qRT-PCR and microarray analysis (n = 3). The vertical axis shows the percentage of each circRNA detected by RT-qPCR and microarray respectively.

Candidate circRNAs were further validated by RT-qPCR; the results are shown in **Figure 7**. The expression levels of mmu-circRNA-010022, mmu-circRNA-008436, and mmu-circRNA-013703 were dysregulated in the GE group in contrast with the control group, which was in accordance with those detected by microarray analysis (**Figure 7**). However, the expression levels of mmu-circRNA-23433 and mmu-circRNA-40996 were inconsistent with the microarray results, that is, their expression levels were upregulated (**Figure 7**). According to a gene symbol, which was closely related to the study, and targeted miRNAs, whose predictive values were less than 1,000, we chose mmu-circRNA-013703 and mmu-circRNA-010022 for further research. Based on the original microarray analysis, the genomic locus of mmu-circRNA-013703/mmu-circRNA-010022 was on chr11/chr19, and the predicted sequence of the best transcript was NM-001024205/NM-013541.

### Construction of circRNA-miRNA-mRNA Co-Expression Network

**Figure 8:**
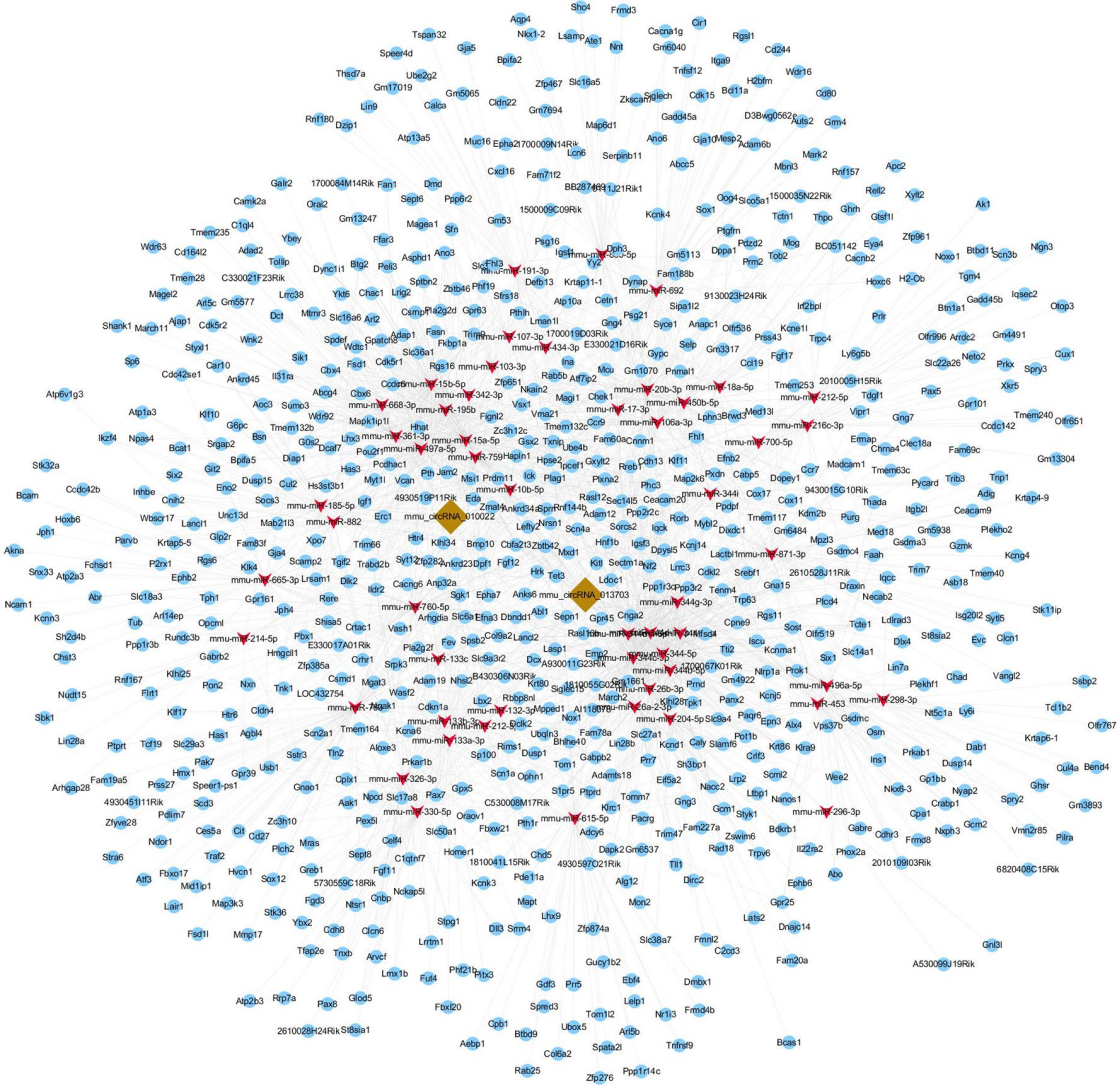
circRNA-miRNA-mRNA co-expression network. The co-expression network was constructed by Cytoscape software based on conjoint analysis of circRNA and mRNA microarray results. Red nodes represent miRNAs, light-blue nodes represent mRNAs, and brown nodes represent circRNAs. The interconnected nodes represent direct relationships.

To further explore the potential functions of circRNA, circRNA and mRNA microarrays were applied to analyze the internal relationships between circRNA, miRNA, and mRNA. In order to obtain the differentially expressed mRNAs targeted by miRNAs, three miRNA databases were used to predict the targeted genes of miRNA. Thereafter, the predicted results were compared and analyzed with the differentially expressed genes in the mRNA microarray data. Such an experiment could clarify the relationship between circRNA, miRNA, and mRNA. We then constructed a circRNA-targeted miRNA-mRNA co-expression network based on the circRNA and mRNA microarray results. The network was composed of 2 circRNAs, 52 miRNAs, and 752 mRNAs (**Figure 8**). The molecules at the heart of the network were the two circRNAs, which radiated to their respective predicted miRNAs (such as miR-361-3p, miR-15a-5p, miR-15b-5p, miR-296-3p, miR-185-5p and so on). Furthermore, the miRNAs were connected to their respective targeted mRNAs. Thus, a co-expression network model for circRNAs regulating targeted miRNAs and miRNAs regulating targeted mRNAs was formed. According to our results, the three largest miRNAs linked by mRNA were mmu-miR-361-3p, mmu-miR-15a-5p, and mmu-miR-15b-5p, of which mmu-miR-361-3p had 80 targeted mRNAs. The results suggested that one differentially expressed circRNA may result in abnormal expression of related mRNAs because they affect miRNAs.

### GO and KEGG Analysis of Circrna Co-Expression Genes

**Figure 9:**
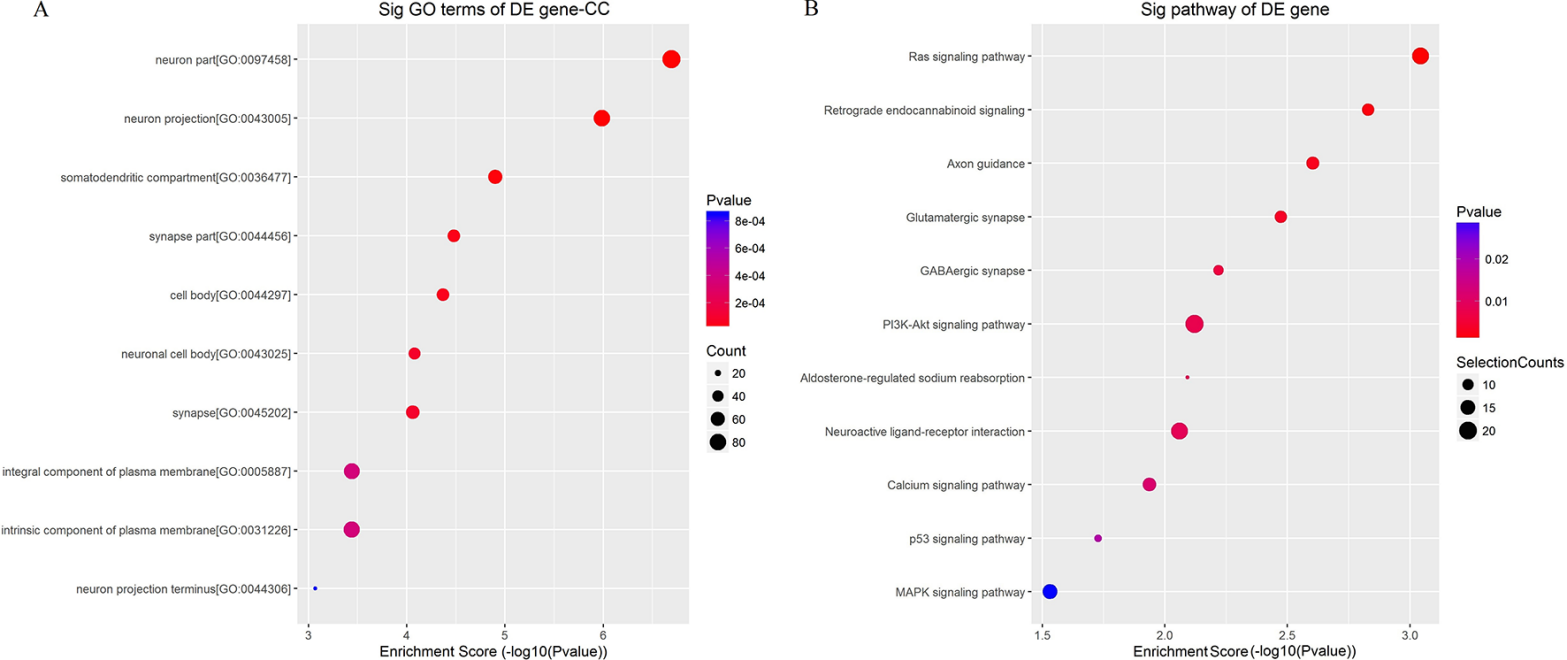
Enrichment analysis of **(A)** GO term and **(B)** pathway term for circRNA co-expression genes. The top 10 changed GO/pathway terms were drawn in enrichment bubble diagrams. Bubble scales represent the number of differently expressed genes. The depth of the bubble color represents the p-value.

To better understand the mechanisms involved in acute GE poisoning, GO enrichment and KEGG pathway analysis were used to explore the functional roles of the circRNA co-expression genes. Go enrichment analysis revealed that 59 MF terms, 38 CC terms, and 379 BP terms were downregulated (P < 0.05). The top 10 generally changed GO terms ranked by enrichment score or p-value, CC was used as an example here, are presented in **Figure 9A**. The results showed that most enriched CC terms were strongly associated with neurons, somatodendritics and synapses [such as “neuron part” (GO: 0097458), “neuron projection” (GO: 0043005), “somatodendritic compartment” (GO: 0036477), “synapse part” (GO: 0044456), and “cell body” (GO: 0044297)].

KEGG pathway analysis was conducted to characterize the targeted genes (**Figure 9B**). The targeted genes were predicted to be closely related with cell survival/demise-related pathways (such as Ras signaling pathway, PI3K-Akt signaling pathway, p53 signaling pathway, and MAPK signaling pathway), neuron/synapse-related pathways (such as retrograde endocannabinoid signaling, axon guidance, glutamatergic synapse, GABAergic synapse, and neuroactive ligand-receptor interaction), and channel-related pathways (such as calcium signaling pathway and aldosterone-regulated sodium reabsorption).

### Functional Module of circRNA-miRNA-mRNA Co-Expression Network

**Figure 10:**
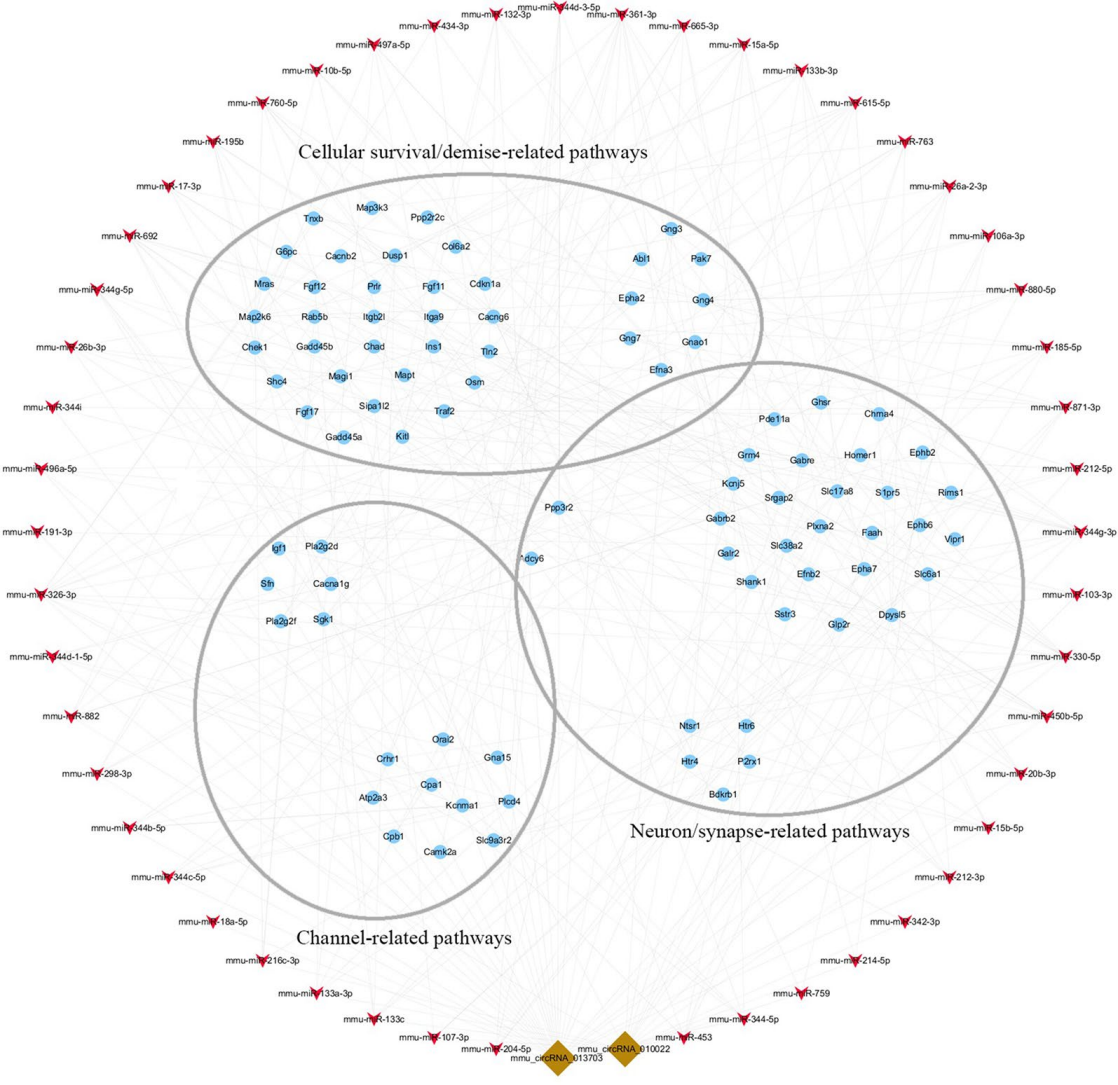
The key function module of circRNA-miRNA-mRNA network. The network was drawn based on the biological function of circRNA co-expression genes. Nodes in red are miRNAs, nodes in light-blue are mRNAs, and nodes in brown are circRNAs. The interconnected nodes represent direct relationships.

To clarify the role of circRNA in acute GE poisoning and to explore the key of its regulatory processes, genes in the aforementioned pathways were selected separately. Thereafter, a new network of three functional modules was formed with circRNA and miRNA (**Figure 10**). Some miRNAs were connected with very few genes. For example, mmu-miR-668-3p only connected six mRNAs, while some miRNAs linked to dozens of genes, among which mmu-miR-361-3p, mmu-miR-15a-5p, and mmu-miR-15b-5p linked to the most mRNAs. This suggested that we could focus on the function of circRNAs and mRNAs associated with miR-361-3p, miR-15a-5p, and miR-15b-5p.

## Discussion

GE is a plant that shows high toxicity and is well-known throughout the world. It is toxic to the digestive, respiratory, and circulatory systems, and even more so to the nervous system (Rujjanawate et al., 2003). The pathomorphological changes in vivo in our earlier experiments on acute toxicity confirmed that GE causes damage to the heart, liver, etc (**Supplementary Figure 3**).

 In this study, mice in the GE group developed neurological symptoms including movement disorders, spasms, and paroxysmal convulsions. As has been previously reported, the neurotoxic mechanism might be related to phase I metabolic enzymes (Wang et al., 2015), γ-ganimalonum (Rujjanawate et al., 2003), etc. Of particular interest in our previous studies of GE was also the involvement of CYP450 and GST in its metabolism (Wang et al., 2018). The liver, as an important organ of metabolism and excretion, is frequently involved with the mechanisms of detoxification/intoxication. However, traditional research models, in which the complex process of intoxication occurrence and development is explained by one or more gene disorders, cannot accurately explain the regulatory mechanisms of intoxication. In recent years, developments have been made on RNA sequencing technologies, which have enabled researchers to conduct systematic analyses on the molecular regulatory mechanisms of thousands of genes involved in diseases. Thus, RNA sequencing technologies were used in our study to comprehensively explore the co-regulation patterns of circRNAs during GE intoxication.

As newfound ncRNAs, circRNAs primarily come from the exons of protein-coding genes *via* nonlinear reverse splicing (Starke et al., 2015). Compared with other ncRNAs (lncRNAs or miRNAs), circRNAs are stable, substantial, and specific to mammalian cells (Memczak et al., 2013). Because of these properties, circRNAs may be ideal molecular biomarkers and potential treatment targets for many diseases (Rybak-Wolf et al., 2015; Chen et al., 2017b). Recently, dysregulated expression of circRNAs has been confirmed to be correlated with nervous system disease, cancer, etc (Lu and Xu, 2016; Wang et al., 2017b). Studies of circRNAs have revealed that they have more miRNA binding sites and more effects on sequestering miRNAs (Wilusz and Sharp, 2013; Guo et al., 2014). Thus, circRNA may remove the inhibitory effect of a miRNA on its targeted gene, raise the expression of the targeted gene, and have a pivotal role in the occurrence and development of diseases (Salmena et al., 2011; Ghosal et al., 2013).

However, to our knowledge, there are no reports on the expression profiles of circRNAs during drug intoxication. Based on the previously mentioned correlation of circRNA-miRNA-targeted gene, circRNA/mRNA microarrays were respectively performed to examine the expression profiles of circRNAs/mRNAs under GE intoxication and normal conditions. Our study first confirmed the changes in expression of circRNAs during GE intoxication in mice. Microarray analysis found that circRNAs (n = 143) and mRNAs (n = 1,921) were significantly expressed (P < 0.05). The dysregulated expression of mmu-circRNA-013703 and mmu-circRNA-010022 were validated in line with the sequencing results using qRT-PCR (**Figure 7**). The results showed that expression levels were affected by GE intoxication. Accordingly, we hypothesized that mmu-circRNA-013703 and mmu-circRNA-010022 might be new molecular biomarkers and play a crucial role in GE intoxication.

For further clarification about the role of mmu-circRNA-013703 and mmu-circRNA-010022, the top 5 putative targeted miRNAs were identified for each circRNA (**Table 2**), and the circRNA-targeted miRNA-mRNA co-expression network was constructed based on the circRNA and mRNA microarray results (**Figure 8**). Essentially, this co-expression network diagramed a cellular RNA network composed of 2 circRNAs interacting with 52 miRNAs and 752 mRNAs (**Figure 8**). This formed a co-expression network model for circRNAs regulating targeted miRNAs and miRNAs regulating targeted mRNAs. Our co-expression network was the principal method by which we predicted the functions of the circRNAs. However, we found that a number of mRNAs might be involved in this co-expression network. Therefore, we investigated the functions of the targeted genes by GO enrichment and KEGG pathway analysis. According to our annotations, the most significant GO terms were neuron, synapse, channel, and transport, indicating that the targeted genes were involved in these major components during GE intoxication. KEGG pathway analysis (**Figure 9**) revealed that the targeted genes were predicted to be closely related with cellular survival/demise-related, neuron/synapse-related, and channel-related pathways. For instance, the PI3K-Akt signaling pathway was reported to be related to cellular survival (Chen et al., 2018a) and the Ras/Raf/ERK1/2 signaling pathway was connected with neurons/synapses (Xu et al., 2016). In line with previous research mechanisms and the neurotoxic symptoms of GE, our GO and KEGG pathway analysis confirmed that neuron/synapse/demise were vital factors for the development of intoxication. The two circRNAs and their potential mechanisms were worthy of further investigation as miRNA inhibitors.

Accordingly, targeted genes in the aforementioned pathways were selected separately. A new network of three functional modules was formed (**Figure 10**) in which miR-361-3p, miR-15a-5p, and miR-15b-5p linked to the most mRNAs. This suggested that we could focus on the function of mmu-circRNA-013703 and mRNAs associated with miR-361-3p, miR-15a-5p, and miR-15b-5p. Prior research has demonstrated that dysregulated miR-361-3p expression results in nonylphenol-induced reproductive toxicity (Tang et al., 2017). MiR-15a-5p was found to be involved in cell survival and apoptosis (Chen et al., 2017a; Chen and Tian, 2017; Zhou et al., 2018). In addition, dysregulated miR-15b-5p is also related to cellular toxicity (de Freitas et al., 2017; Chen et al., 2018b). Thus, we could conclude that the mmu-circRNA-013703 is involved in GE intoxication by sponging intoxication-related miRNAs. Among the targeted genes regulated by miR-361-3p, the following have been reported to be connected with cellular survival/demise according to PubMed (https://www.ncbi.nlm.nih.gov/pubmed/): Wnk2, Cdkn1a, Crhr1, Hrk, Ick, Map2k6, Mxd1, Npas4, Shisa5, and Txnip. Anp32a, Camk2a, Magel2, Shank1, and Syt12 were relevant to neurons/synapses. As for miR-15a-5p and miR-15b-5p, their targeted genes were basically the same except Tln2 (regulated only by miR-15a-5p) and Pcdhac1 (regulated only by miR-15b-5p). Similarly, targeted genes associated with cellular survival/demise (Sgk1, Lrig2, etc.) and targeted genes associated with neuron/synapse (Chac1, Myt1l, etc.) were found to be related with miR-15a-5p and miR-15b-5p. However, some of the targeted genes, such as Wdr63, Tmem132b, and Psg16, so far appear to have no functions associated with GE intoxication. The nature of their roles needs to be resolved. Furthermore, the regulatory mechanisms of mmu-circRNA-013703 still remain to be elucidated. In short, this study gave further support to the hypothesis that mmu-circRNA-013703 functions as a sponge of miRNAs to regulate the more comprehensive circRNA-miRNA-mRNA co-expression network.

In conclusion, our circRNA/mRNA microarray analysis presented a landscape of dysregulated circRNAs/mRNAs during GE intoxication. Two circRNAs were confirmed to be aberrantly expressed during GE intoxication. A co-expression network was constructed for circRNA-miRNA-mRNA. Further investigation demonstrated that mmu-circRNA-013703 might sponge intoxication-related miRNAs and thus affect their targeted gene expression, implying its involvement in intoxication and its potential use as a biomarker and target for the prevention of intoxication. Our results may hopefully stimulate other researchers to take drug intoxication more seriously.

## Data Availability Statement

The datasets generated for this study can be found in the GEO Using the accession number: GPL21826.

## Ethics Statement

All experimental procedures complied with international ethical guidelines and the Guide for the Care and Use of Laboratory Animals (National Institutes of Health). The animal protocol was approved by the Ethics Committee of Fujian University of Traditional Chinese Medicine (FJTCM [2018] Ethics Review No. 018)

## Author Contributions

YW and SW contributed toward conception and design of research. CY, HG, and HY performed experiments. SW interpreted the results of the experiments. YW, XL, and SY analyzed the data. YW drafted the manuscript. All the authors listed have approved the manuscript.

## Funding

This work was supported by the National Natural Science Foundation of China (Grant No. 81773921 and 81303240).

## Conflict of Interest

The authors declare that the research was conducted in the absence of any commercial or financial relationships that could be construed as a potential conflict of interest.
