# Supplementary material for: Construction and Analysis of circRNA-miRNA-mRNA Molecular Regulatory Networks During Herba Gelsemium elegans Intoxication
Source: Front Pharmacol. 2019 Oct 17;10:1217. doi: 10.3389/fphar.2019.01217 (PMC6812611; doi:10.3389/fphar.2019.01217)
Supplement: Supplementary file 1 [file DataSheet_1.pdf]

Fig.S1

mmu_circRNA_23433 VS mmu-miR-7242-5p

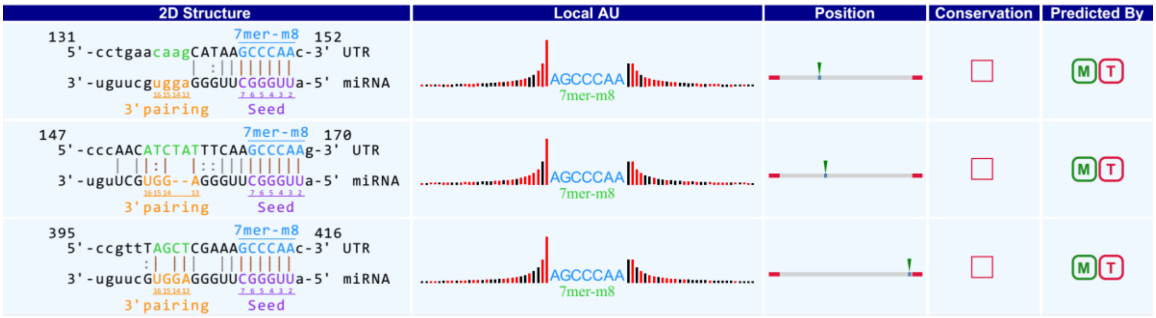

mmu_circRNA_008436 VS mmu-miR-1934-5p

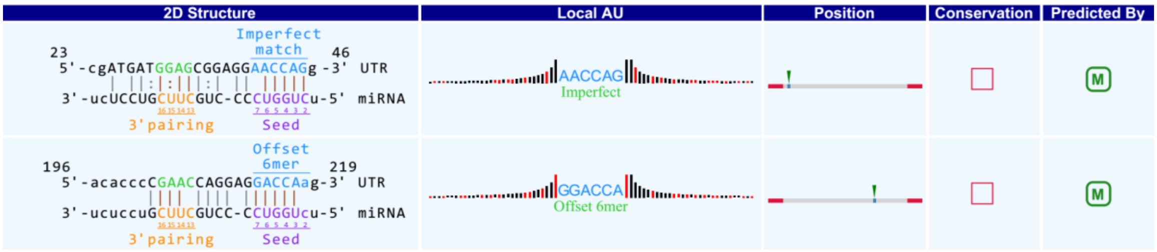

mmu_circRNA_40996 VS mmu-miR-344d-2-5p

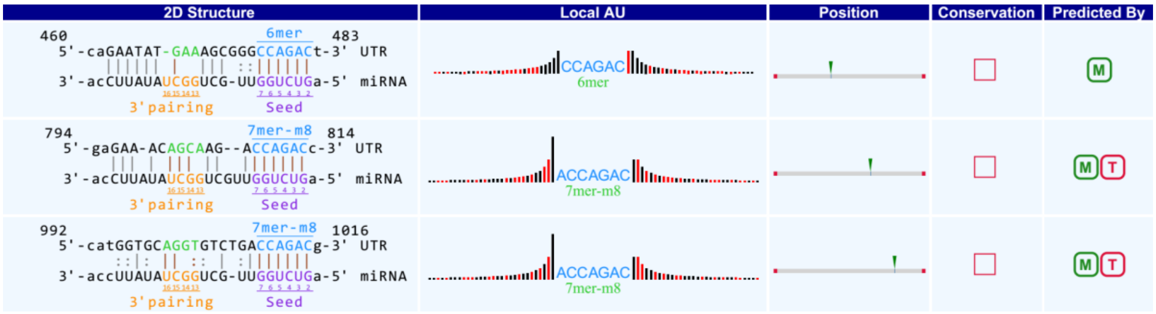

Fig.S1 Binding site of mmu_circRNA_23433 VS mmu-miR-7242-5p, mmu_circRNA_008436 VS mmu-miR-1934-5p and mmu_circRNA_40996 VS mmu-miR-344d-2-5p. The upper part is the sequence at the junction of circRNA and the lower part is the sequence of miRNA.

Fig.S2

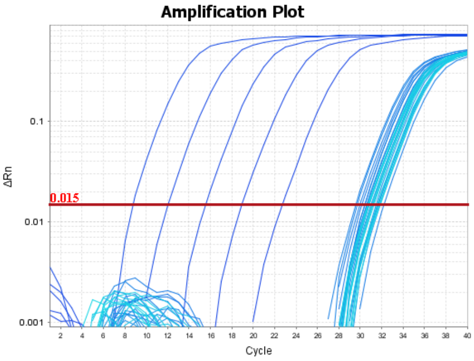

A$_1$ Amplification Plot

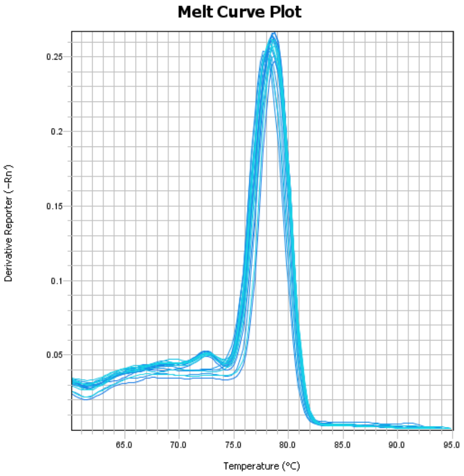

A$_2$ Melt Curve Plot

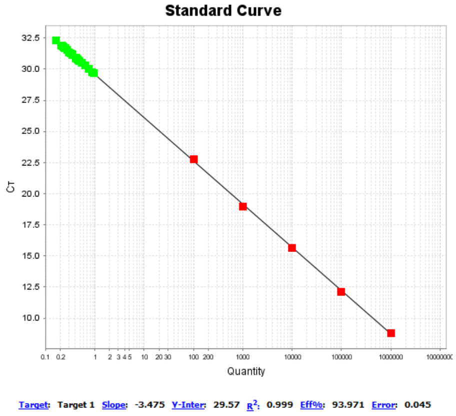

A$_3$ Standard Curve

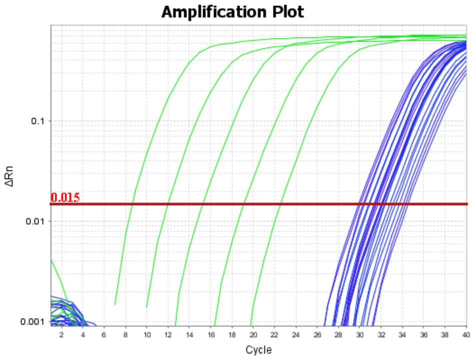

B$_1$ Amplification Plot

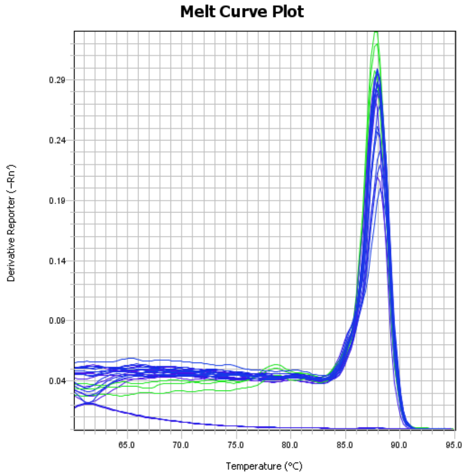

B$_2$ Melt Curve Plot

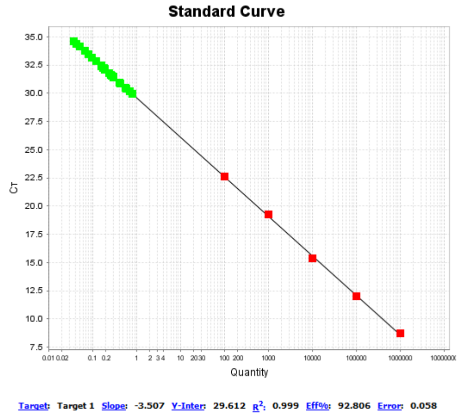

B$_3$ Standard Curve

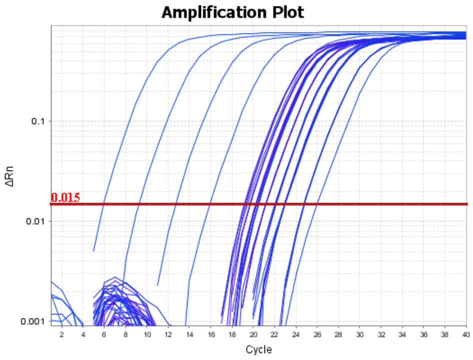

C$_1$ Amplification Plot

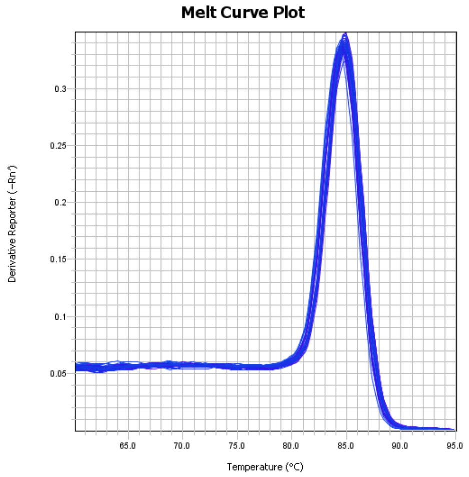

C$_2$ Melt Curve Plot

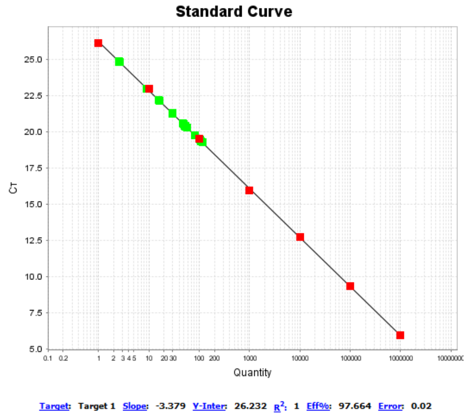

C$_3$ Standard Curve

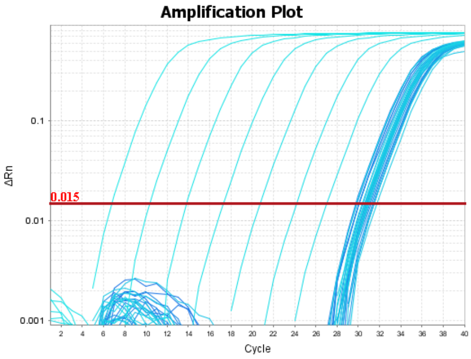

D$_1$ Amplification Plot

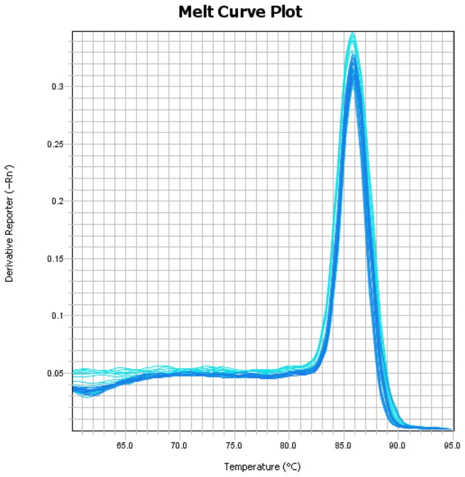

D$_2$ Melt Curve Plot

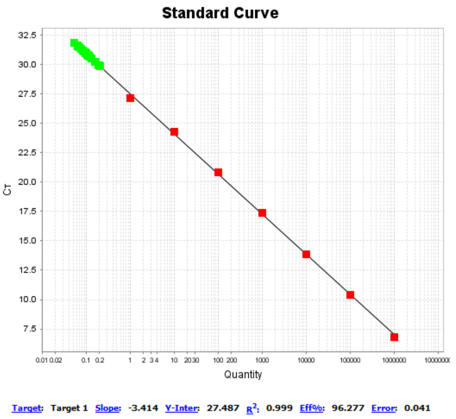

D$_3$ Standard Curve

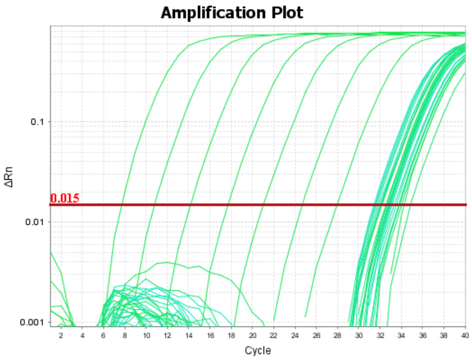

E$_1$ Amplification Plot

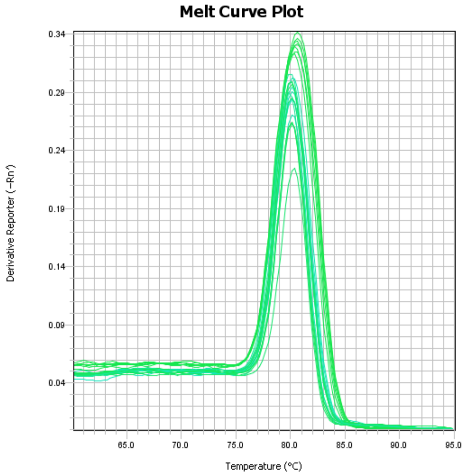

E$_2$ Melt Curve Plot

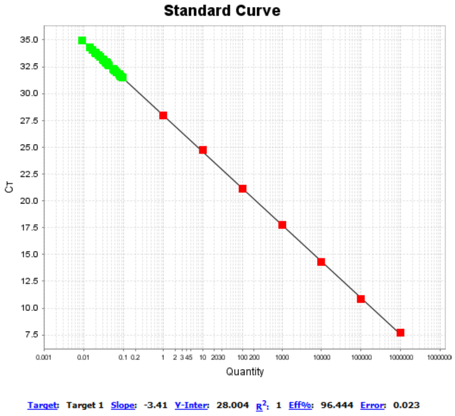

E$_3$ Standard Curve

Fig.S2 Amplification plot, melt curve plot and standard curve of five circRNAs. A, mmu-circRNA-013703; B, mmu-circRNA-008436; C, mmu-circRNA-010022; D, mmu-circRNA-23433; E, mmu-circRNA-40996

Fig.S3

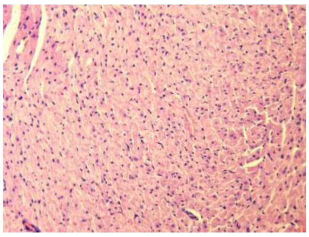

A Heart (HE×200)

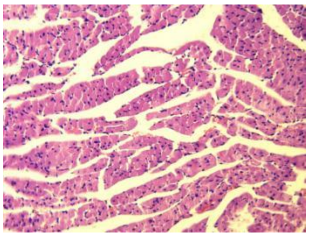

B Heart (HE×200)

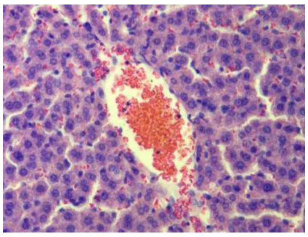

A Liver (HE×400)

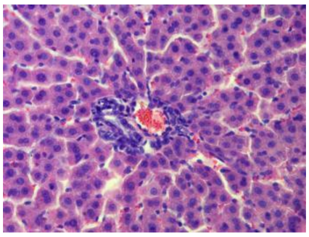

B Liver (HE×400)

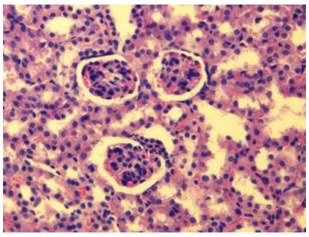

A Kidneys (HE×400)

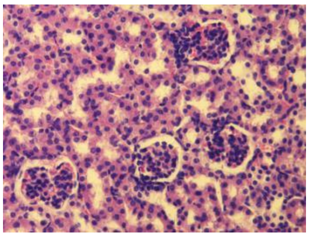

B Kidneys (HE×400)

Fig.S3 Morphological changes during GE intoxication. Groups: A, control group; B, GE group

Fig.S4

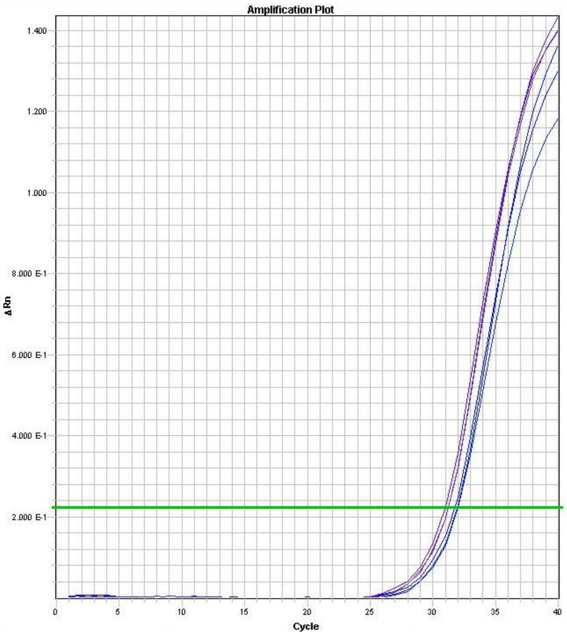

ADCY6 Amplification Plot

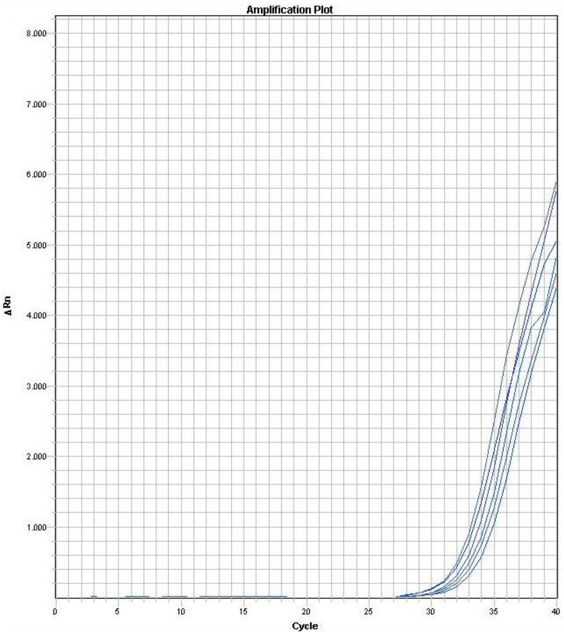

ATP1A3 Amplification Plot

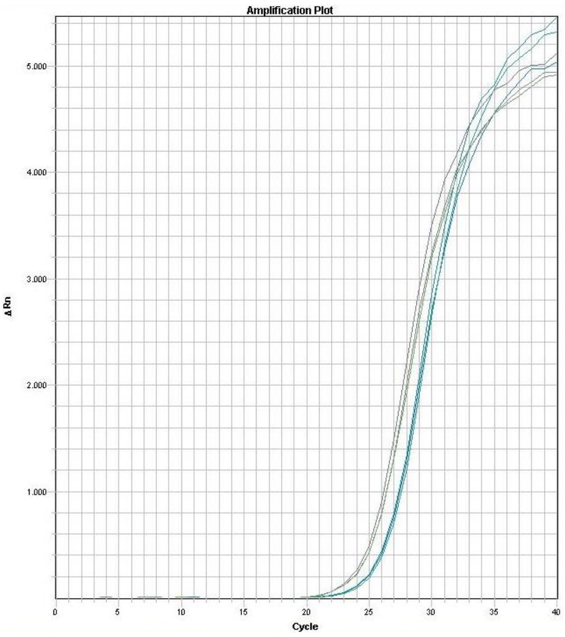

EPHA2 Amplification Plot

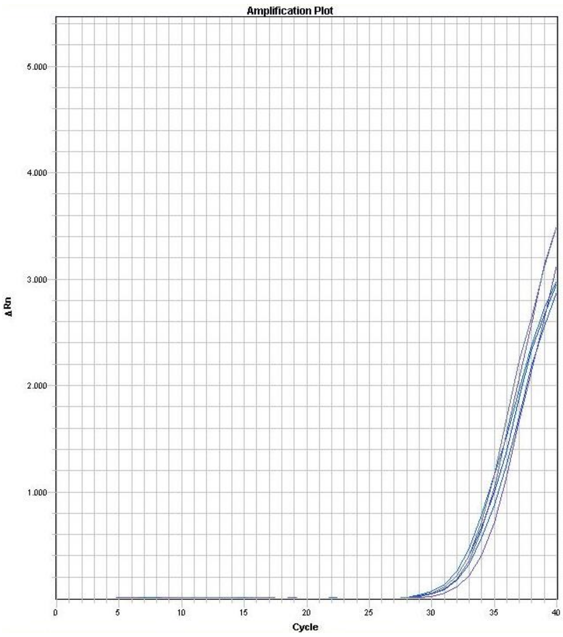

GNG3 Amplification Plot

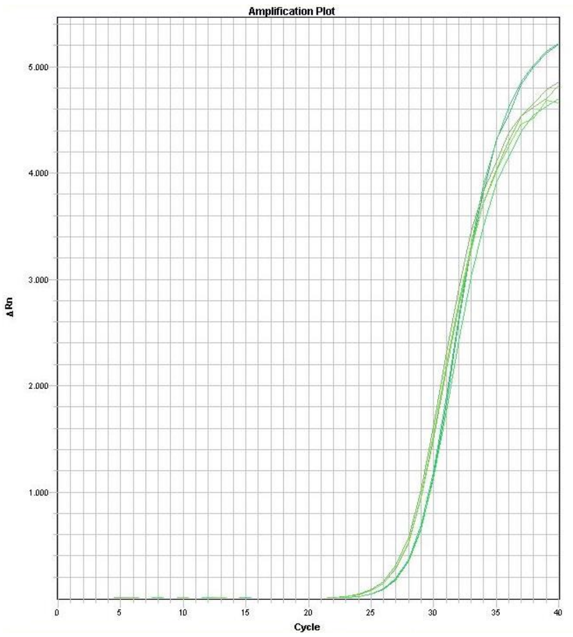

KITL Amplification Plot

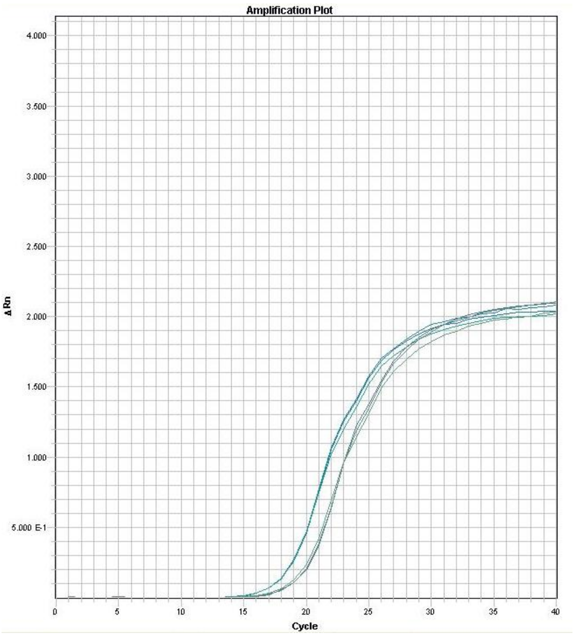

IGF1 Amplification Plot

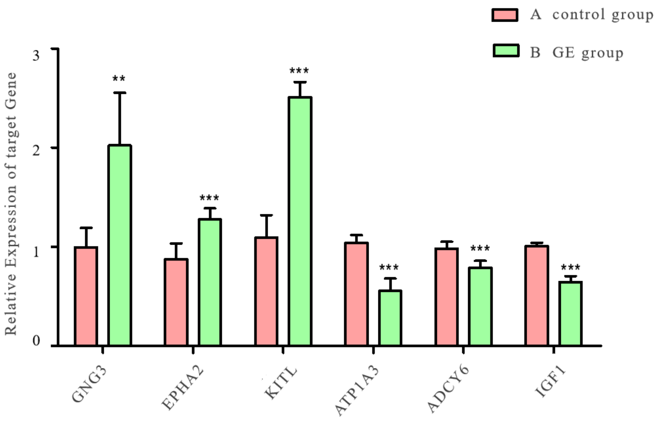

Relative expression of target genes

Fig.S4 Amplification Plot and relative expression of ADCY6, ATP1A3, EPHA2, GNG3, KITL and IGF1. *P ＜0.05, **P＜0.01, ***P＜0.001, versus control

Supplementary Table 1

Supplementary Table 1 Other significantly up/downregulated circRNAs and Gene Symbol

circRNA	Regulation	*P*-value	fold change	circRNA_type	Chromosome	Best_transcript	Gene Symbol
mmu_circRNA_27842	up	0.02660	1.78	exonic	chr14	NM_001164503	Akap11
mmu_circRNA_19495	up	0.01453	1.53	sense overlapping	chr8	NM_199446	Phkb
mmu_circRNA_22927	up	0.04467	1.89	exonic	chr11	NM_146243	Actr2
mmu_circRNA_42366	up	0.02396	1.74	exonic	chr7	NM_010207	Fgfr2
mmu_circRNA_012180	up	0.02486	1.54	exonic	chr8	NM_199446	Phkb
mmu_circRNA_23433	up	0.02785	1.55	exonic	chr11	NM_021354	Drg2
mmu_circRNA_30663	up	0.01031	2.03	exonic	chr17	NM_175333	Slc25a41
mmu_circRNA_21079	up	0.03082	1.58	exonic	chr1	NM_001136104	Abl2
mmu_circRNA_007735	up	0.00695	1.85	exonic	chr19	NM_145505	Fam160b1
mmu_circRNA_001644	up	0.01177	1.57	exonic	chr7	NM_207302	Zranb1
mmu_circRNA_43389	up	0.01584	1.68	exonic	chr8	NM_022309	Cbfb
mmu_circRNA_35148	up	0.04084	1.76	exonic	chr3	NM_030732	Tbl1xr1
mmu_circRNA_26114	up	0.02150	1.95	exonic	chr13	NM_027118	Cdk13
mmu_circRNA_28609	up	0.02135	1.97	exonic	chr15	NM_145470	Deptor
mmu_circRNA_21054	up	0.02441	1.54	exonic	chr1	NM_028250	Acbd6
mmu_circRNA_008057	up	0.04043	1.71	exonic	chr9	NM_144937	Usp3
mmu_circRNA_014539	up	0.03401	1.71	exonic	chr7	NM_181517	Ipo7
mmu_circRNA_19525	up	0.04766	1.64	sense overlapping	chr9	NM_144937	Usp3
mmu_circRNA_36616	up	0.02640	1.57	exonic	chr4	NM_011884	Rngtt
mmu_circRNA_31512	down	0.02760	2.31	exonic	chr18	NM_001110015	Wdr36

mmu_circRNA_015474	down	0.03714	1.76	antisense	chr19	NM_010123	Eif3a
mmu_circRNA_20709	down	0.04657	1.90	exonic	chr1	NM_019933	Ptpn4
mmu_circRNA_30235	down	0.02387	1.56	exonic	chr17	NM_011948	Map3k4
mmu_circRNA_41362	down	0.00742	1.54	exonic	chr7	NM_001112739	Kcnc1
mmu_circRNA_20282	down	0.00859	3.62	exonic	chr1	NM_175510	Unc80
mmu_circRNA_19307	down	0.01812	1.85	sense overlapping	chr3	NM_010259	Gbp2b
mmu_circRNA_36357	down	0.04539	1.97	exonic	chr3	NM_001093752	Srsf11
mmu_circRNA_33285	down	0.04251	1.76	exonic	chr2	NM_172661	Prrc2b
mmu_circRNA_45756	down	0.03223	3.26	exonic	chrX	NM_177750	Frmpd3
mmu_circRNA_42064	down	0.00103	1.56	exonic	chr7	NR_037617	St5
mmu_circRNA_27840	down	0.03327	2.91	sense overlapping	chr14	NM_025384	Dnajc15
mmu_circRNA_34136	down	0.03290	1.52	exonic	chr2	NM_001033347	D430041D05Rik
mmu_circRNA_43430	down	0.03308	1.54	exonic	chr8	NM_018823	Nfat5
mmu_circRNA_38638	down	0.04933	2.07	intergenic	chr5		
mmu_circRNA_34796	down	0.04687	1.80	sense overlapping	chr2	ENSMUST00000118786	Gm14494
mmu_circRNA_41421	down	0.00801	1.84	exonic	chr7	NM_080853	Slc17a6
mmu_circRNA_23254	down	0.00811	1.56	exonic	chr11	NM_001290709	Ebf1
mmu_circRNA_23074	down	0.03717	1.56	exonic	chr11	NM_027260	Vrk2
mmu_circRNA_25572	down	0.04618	2.00	exonic	chr12	NM_144524	Angel1
mmu_circRNA_33280	down	0.01057	1.52	exonic	chr2	NM_172661	Prrc2b
mmu_circRNA_25827	down	0.00576	1.54	intronic	chr12	uc007pat.2	Meg3
mmu_circRNA_24650	down	0.04298	1.98	sense overlapping	chr12	ENSMUST00000175951	Gm24758

mmu_circRNA_009247	down	0.03089	1.75	exonic	chr19	NM_198300	Cpeb3
mmu_circRNA_22351	down	0.04381	1.60	exonic	chr10	NM_026070	Ccdc53
mmu_circRNA_31342	down	0.04437	1.54	exonic	chr18	NM_145492	Zfp521
mmu_circRNA_33211	down	0.04367	1.66	exonic	chr2	NM_009442	Ttf1
mmu_circRNA_014264	down	0.02483	2.72	antisense	chr6	NM_008973	Ptn
mmu_circRNA_34416	down	0.03638	2.26	exonic	chr2	NM_178795	Ppip5k1
mmu_circRNA_19219	down	0.00222	2.01	sense overlapping	chr19	NM_013456	Actn3
mmu_circRNA_25595	down	0.00354	1.78	exonic	chr12	NM_172544	Nrxn3
mmu_circRNA_42497	down	0.02943	1.55	exonic	chr7	NM_001113373	Shank2
mmu_circRNA_28644	down	0.03466	1.58	exonic	chr15	NM_010026	Asap1
mmu_circRNA_43904	down	0.04378	1.85	exonic	chr9	NM_001033323	Igsf9b
mmu_circRNA_33317	down	0.00215	1.54	exonic	chr2	NM_175184	Mvb12b
mmu_circRNA_35476	down	0.03643	2.11	exonic	chr3	NM_020007	Mbnl1
mmu_circRNA_34815	down	0.00493	1.83	exonic	chr2	NM_001004721	Pigu
mmu_circRNA_017967	down	0.04749	2.49	antisense	chr1	NM_009930	Col3a1
mmu_circRNA_29304	down	0.04305	1.66	exonic	chr16	NM_011159	Prkdc
mmu_circRNA_34224	down	0.02464	1.73	intronic	chr2	ENSMUST00000003705	Aven
mmu_circRNA_45965	down	0.03967	1.61	exonic	chrX	NM_183427	Glra2
mmu_circRNA_43180	down	0.00465	1.68	exonic	chr8	NM_011925	Adgre5
mmu_circRNA_43151	down	0.02360	2.04	exonic	chr8	NM_008539	Smad1
mmu_circRNA_23571	down	0.04410	1.54	antisense	chr11	NM_175681	Glp2r
mmu_circRNA_41215	down	0.04247	1.90	antisense	chr7	NM_009696	Apoe

mmu_circRNA_21271	down	0.03445	1.86	exonic	chr1	NM_025321	Sdhc
mmu_circRNA_42212	down	0.03661	1.91	exonic	chr7	NM_027815	9030624J02Rik
mmu_circRNA_24475	down	0.01608	1.57	exonic	chr11	NM_028898	Rptor
mmu_circRNA_23299	down	0.02664	1.66	exonic	chr11	NM_001294323	Zfp2
mmu_circRNA_31801	down	0.00921	1.78	exonic	chr18	NM_134134	Hmgxb3
mmu_circRNA_31315	down	0.03435	1.84	exonic	chr18	NM_001190371	Ankrd29
mmu_circRNA_22793	down	0.04579	1.91	exonic	chr10	NM_019963	Stat2
mmu_circRNA_39113	down	0.01868	1.53	exonic	chr5	NM_011578	Tgfbr3
mmu_circRNA_43475	down	0.02231	2.30	sense overlapping	chr8	NM_198308	Pdpr
mmu_circRNA_002967	down	0.04555	1.62	exonic	chr2	NM_010899	Nfatc2
mmu_circRNA_30883	down	0.02505	1.51	exonic	chr17	NM_027864	Galnt14
mmu_circRNA_010231	down	0.04084	1.80	exonic	chr12	NM_016893	Fut8
mmu_circRNA_38586	down	0.01774	1.89	exonic	chr5	NM_133911	Adgra2
mmu_circRNA_35586	down	0.01429	1.73	intronic	chr3	AK044425	
mmu_circRNA_20613	down	0.00059	1.51	exonic	chr1	NM_013784	Pign
mmu_circRNA_23516	down	0.00541	2.14	sense overlapping	chr11	NM_133208	Zfp287
mmu_circRNA_33540	down	0.03356	2.23	exonic	chr2	NM_133839	Mmadhc
mmu_circRNA_004280	down	0.02595	1.60	exonic	chr9	ENSMUST00000183955	Mlip
mmu_circRNA_19629	down	0.01410	2.14	exonic	chr1	NM_177834	Cpa6
mmu_circRNA_24338	down	0.03513	1.89	exonic	chr11	NM_176850	Bptf
mmu_circRNA_26077	down	0.04183	1.93	exonic	chr13	NM_001081348	Hecw1
mmu_circRNA_30470	down	0.03012	1.81	sense overlapping	chr17	NM_172458	Zfp871

mmu_circRNA_19397	down	0.01848	1.81	sense overlapping	chr5	NM_175362	Card11
mmu_circRNA_30780	down	0.03119	2.06	exonic	chr17	NM_009067	Ralbp1
mmu_circRNA_39082	down	0.03696	1.74	intronic	chr5	ENSMUST00000112707	Lrrc8b
mmu_circRNA_015178	down	0.04468	1.50	exonic	chr12	NM_172803	Dock4
mmu_circRNA_22752	down	0.01869	1.52	exonic	chr10	NM_176919	Ppm1h
mmu_circRNA_29082	down	0.04534	1.84	exonic	chr15	NM_134093	Letmd1
mmu_circRNA_23589	down	0.01234	1.88	exonic	chr11	NM_011065	Per1
mmu_circRNA_30543	down	0.04012	1.95	sense overlapping	chr17	NM_009358	Ppp2r5d
mmu_circRNA_22359	down	0.04586	1.62	exonic	chr10	NM_001004164	Gnptab
mmu_circRNA_41475	down	0.03530	1.72	exonic	chr7	NM_011668	Ube3a
mmu_circRNA_41278	down	0.02558	1.51	exonic	chr7	NM_019546	Prodh2
mmu_circRNA_27157	down	0.04733	2.23	exonic	chr14	NM_009674	Anxa7
mmu_circRNA_35239	down	0.03933	1.88	exonic	chr3	NM_021483	Pex5l
mmu_circRNA_23602	down	0.02892	2.33	exonic	chr11	NM_007864	Dlg4
mmu_circRNA_32233	down	0.01236	2.49	exonic	chr19	NM_172302	Cpsf7
mmu_circRNA_33193	down	0.03682	1.73	exonic	chr2	NM_013678	Surf2
mmu_circRNA_20363	down	0.00478	1.78	exonic	chr1	NM_007463	Speg
mmu_circRNA_26419	down	0.04519	1.66	sense overlapping	chr13	NM_001025074	Ntrk2
mmu_circRNA_39271	down	0.04210	1.81	exonic	chr5	NM_172722	Naa25
mmu_circRNA_37628	down	0.03296	1.69	exonic	chr4	NM_001114399	Zmym4
mmu_circRNA_21252	down	0.04169	2.22	exonic	chr1	NM_133806	Uap1
mmu_circRNA_20357	down	0.01785	1.62	exonic	chr1	NM_001014974	Ttll4

mmu_circRNA_014583	down	0.02831	2.09	exonic	chr4	NM_018799	Eif3i
mmu_circRNA_39014	down	0.02696	1.51	exonic	chr5	NM_133738	Antxr2
mmu_circRNA_24696	down	0.04833	1.72	sense overlapping	chr12	NM_173417	Kcns3
mmu_circRNA_24213	down	0.04443	1.73	exonic	chr11	NM_183034	Plekhm1
mmu_circRNA_017685	down	0.00097	1.87	intronic	chr16	AK163812	
mmu_circRNA_39660	down	0.04064	1.81	exonic	chr5	NM_029438	Smurf1
mmu_circRNA_31167	down	0.02770	1.51	intergenic	chr17		
mmu_circRNA_017649	down	0.04246	3.02	exonic	chr1	NM_178119	Agap1
mmu_circRNA_42058	down	0.04255	1.60	exonic	chr7	NM_021885	Tub
mmu_circRNA_44291	down	0.04946	1.75	sense overlapping	chr9	NM_009145	Nptn
mmu_circRNA_42995	down	0.01281	1.58	exonic	chr8	NM_172753	Csgalnact1
mmu_circRNA_40634	down	0.01464	1.54	exonic	chr6	ENSMUST00000155466	Gm20696
mmu_circRNA_22603	down	0.04813	1.85	exonic	chr10	NM_001081035	Nav3
mmu_circRNA_40286	down	0.04617	2.05	exonic	chr6	NM_173406	Jazf1
mmu_circRNA_39643	down	0.00205	1.51	sense overlapping	chr5	NM_008886	Pms2
mmu_circRNA_32256	down	0.03484	1.60	sense overlapping	chr19	TCONS_00015994	XLOC_011860
mmu_circRNA_30323	down	0.04386	1.87	exonic	chr17	NM_011647	Tsc2
mmu_circRNA_015001	down	0.04902	1.64	antisense	chr1	NR_002840	Gas5
mmu_circRNA_25690	down	0.00644	1.65	exonic	chr12	NM_001033213	Ttc7b
mmu_circRNA_36086	down	0.01647	1.78	exonic	chr3	NM_172525	Arhgap29
mmu_circRNA_38636	down	0.04815	1.99	sense overlapping	chr5	NM_001122758	Pcdh7
mmu_circRNA_006263	down	0.00059	1.71	sense overlapping	chr10	NM_029881	Tmem200a

mmu_circRNA_43170	down	0.04455	2.00	sense overlapping	chr8	NM_008357	Il15
mmu_circRNA_43816	down	0.01784	1.67	exonic	chr9	NM_173777	Olfm2
mmu_circRNA_34234	down	0.02777	1.68	exonic	chr2	NM_177652	Ryr3
mmu_circRNA_28787	down	0.01399	1.71	sense overlapping	chr15	NM_011117	Plec
mmu_circRNA_008839	down	0.00866	1.50	exonic	chrX	uc009uss.2	Sh3kbp1
mmu_circRNA_25311	down	0.03659	1.80	exonic	chr12	NM_153457	Rtn1
mmu_circRNA_006877	down	0.04031	1.52	exonic	chr1	NM_030724	Uck2
